# Assessing Dispositions Toward Ridicule and Laughter in the Workplace: Adapting and Validating the PhoPhiKat-9 Questionnaire

**DOI:** 10.3389/fpsyg.2017.00714

**Published:** 2017-05-12

**Authors:** Jennifer Hofmann, Willibald Ruch, René T. Proyer, Tracey Platt, Fabian Gander

**Affiliations:** ^1^Personality and Assessment, Department of Psychology, University of ZurichZurich, Switzerland; ^2^Swiss National Centre of Competence in Research Lives–Overcoming Vulnerability: Life Course PerspectivesLausanne, Switzerland; ^3^Department of Psychology, Martin-Luther University Halle-WittenbergHalle, Germany; ^4^Institute of Psychology, University of WolverhamptonWolverhampton, UK

**Keywords:** assessment, bullying, gelotophobia, humor, laughter, work satisfaction, work place

## Abstract

The current paper addresses the measurement of three dispositions toward ridicule and laughter; i.e., gelotophobia (the fear of being laughed at), gelotophilia (the joy of being laughed at), and katagelasticism (the joy of laughing at others). These traits explain inter-individual differences in responses to humor, laughter, and social situations related to humorous encounters. First, an ultra-short form of the PhoPhiKat-45 (Ruch and Proyer, 2009) was adapted in two independent samples (Construction Sample *N* = 157; Replication Sample *N* = 1,774). Second, we tested the validity of the PhoPhiKat-9 in two further independent samples. Results showed that the psychometric properties of the ultra-short form were acceptable and the proposed factor structure could be replicated. In *Validation Sample 1* (*N* = 246), we investigated the relation of the three traits to responses in a ridicule and teasing scenario questionnaire. The results replicated findings from earlier studies by showing that gelotophobes assigned the same emotions to friendly teasing and malicious ridicule (predominantly low joy, high fear, and shame). Gelotophilia was mainly predicted by relating joy to both, teasing and ridicule scenarios, while katagelasticism was predicted by assigning joy and contempt to ridicule scenarios. In *Validation Sample 2* (*N* = 1,248), we investigated whether the fear of being laughed at is a vulnerability at the workplace: If friendly teasing and laughter of co-workers, superiors, or customers are misperceived as being malicious, individuals may feel less satisfied and more stressed. The results from a representative sample of Swiss employees showed that individuals with a fear of being laughed at are generally less satisfied with life and work and experience more work stress. Moreover, gelotophilia went along with positive evaluations of one's life and work, while katagelasticism was negatively related to work satisfaction and positively related to work stress. In order to establish good work practices and build procedures against workplace bullying, one needs to consider that individual differences impact on a person's perception of being bullied and assessing the three dispositions may give important insights into team processes.

## Introduction

Although humor and laughter are commonly viewed as positively valued, empirical evidence suggests individual differences in the perception of laughter and laughter-related events (see Ruch et al., 2014). Three dispositions toward laughter and ridicule (Ruch and Proyer, 2009) have been coined to define specific inter-individual tendencies to either (a) fearing being laughed at (Ruch and Proyer, 2008a,b; gelotophobia), (b) enjoying being laughed at (gelotophilia; Ruch and Proyer, 2009), or (c) enjoying to laugh at others (katagelasticism; Ruch and Proyer, 2009).

Individuals with a fear of being laughed at display biases in their perception of humor and laughter, as well as in responses to those phenomena (see Ruch et al., 2014). They see humor and laughter as negative, aversive, and directed toward them in a malicious way (see e.g., Ruch and Proyer, 2008a; Ruch et al., 2014). For example, they respond to both, friendly teasing and malicious ridicule with higher felt shame, fear, and low joy in predefined scenarios of ridicule and teasing. They do not emotionally distinguish between the different contexts (Platt, 2008). Gelotophobes screen social interaction partners for signs of derision, and often show paranoid tendencies toward being laughed at. They further display disproportionate negative responses toward anticipated ridicule. Moreover, they respond with controlling themselves and their environment, withdrawing, or internalizing when confronted with (anticipated) ridicule (Papousek et al., 2009; Platt et al., 2012; Ruch et al., 2014). Moreover, gelotophobes experience marked heart rate deceleration when hearing laughter (indicating a “freezing-like” response; Papousek et al., 2014).

Thus, gelotophobes respond to the pro-social bonding and group building aspects of humor and laughter with aversion and misinterpretation, which can have detrimental effects on social interaction. Whereas, withdrawing from fear-evoking situations may be manageable in their personal lives, they will encounter problems in the work place where they presumably cannot avoid engaging in social interactions. It is speculated that gelotophobes will find humorous interactions with (unfamiliar) customers and staff, team members, and supervisors difficult (Ruch et al., 2014): they are likely to misinterpret friendly banter and humor in the work place more often as negative, will screen the environment for laughter and will attribute this laughter as being laughed at. In line with this, Ruch and Proyer (2008b) already predicted that higher degrees of gelotophobia should be found in victims of bullying (e.g., at the workplace see Ruch et al., 2014) and related to phenomena like aggressiveness^1^ or coherence within social groups (see Samson and Meyer, 2010). Additionally, Platt et al. (2009) confirmed that gelotophobia correlated positively with reports of having been a victim of bullying. While this may be distressing for the individual, it has also implications on a broader level too. At the level of organizations, such behaviors could seriously impact on employees' well-being, be a potential financial burden when going along with increased social welfare payments, have an impact on over-stretching health service resources, and potentially add costs to spurious employment ligations.

Nevertheless, these predictions have so far not been substantiated in a working context; i.e., in representative samples of the workforce of a given country. This is relevant, as the perceived bullying and discrimination may be based on “false alarms” due to gelotophobia, while there is actually no objective evidence for it (Ruch and Proyer, 2008a). Such misperceptions may reflect in lower work and life satisfaction (see Proyer et al., 2012b), as well as higher work stress. For the co-workers and supervisors, claims of bullying assaults need to be taken seriously, but they should also take into account the individual differences in the perception of humor and laughter, if other evidence does not corroborate the claims.

While gelotophobes dread the laughter of others, *gelotophiles* actively seek it: They readily tell others of their mishaps and embarrassing situations because they enjoy the laughter of others that these stories elicit (Ruch and Proyer, 2009). They explicitly seek potentially embarrassing situations for the joy of recalling this to an audience. As expected, gelotophobia is negatively correlated to gelotophilia (Ruch and Proyer, 2009). In a work context, it is assumed that gelotophiles will be frequent elicitors of humor and laughter (particularly when it relates to them) and they will perceive friendly banter as joyful. They will be viewed as the “good cheer” of the group. Thus, we hypothesize that gelotophilia will positively related to work and life satisfaction (in line with former findings, see Ruch et al., 2014 for an overview) and negatively to work stress, due to their ability to laugh at their mishaps and ability to initiate humor and laughter.

The third disposition relates to those who experience joy when laughing at others, *katagelasticism* (Ruch and Proyer, 2009). Katagelasticists screen their peers carefully to find instances or causes of amusement. These triggers are then used for making others laugh. They actively search for situations where they can laugh at others and do not feel guilty for doing so. As the saying goes “an eye for an eye, a tooth for a tooth,” their aim is for the targets of their mockery to take revenge and joke or prank back by trying to out perform the initial joke (Ruch and Proyer, 2009). While katagelasticism is positively correlated to gelotophilia, typically no relation to gelotophobia is found. Therefore, some gelotophobes might as well-enjoy laughing at others, whereas others will not. In work place contexts, katagelasticists are predicted to be seen as the “bullies” as they enjoy laughing at others and be the ones who encounter problems in the work place, as they behave socially undesirable by laughing at others frequently.

The three traits can be reliably assessed with a self-report measure, the PhoPhiKat-45 (Ruch and Proyer, 2009). Many studies have shown the reliability and validity (cf. Ruch et al., 2014). The scale allows separating the gelotophobia spectrum (with means ranging from 1 to 4) into groups of no fear (<2.5 on the gelotophobia scale), a slight fear (>2.5), a marked fear (>3.0), and extreme fear of being laughed at (>3.5; see Ruch and Proyer, 2008a). While a 30-item short form (Ruch and Proyer, 2009) exists, an ultra-short version is required for research and application. In research contexts, the short form can be utilized for screening purposes and the use in large-scale studies. In the latter, the number of items for the assessment of constructs is often limited and the comparatively lower reliabilities can be compensated by larger sample sizes. In the applied context, the short form can serve as an economic instrument for the screening of the three dispositions toward laughter in large groups, for work place counseling, and the investigation of team processes (yet, the ultra-short form always needs to be complemented by the long form for individual counseling).

The aims of the current study were two-fold. First, we aimed to develop an ultra-short form of the standard self-report questionnaire on the three dispositions toward ridicule and laughter, the PhoPhiKat-45 (Ruch and Proyer, 2009). This newly developed questionnaire, labeled PhoPhiKat-9 was tested for its psychometric properties^2^. The development of the ultra-short form was motivated by the necessity to include a brief measure of the PhoPhiKat-9 in the project conducted by the Swiss National Centre of Competence in Research (LIVES—Overcoming vulnerability: Life course perspectives), which examines the effects of the post-industrial economy and society on the development of vulnerability (using a longitudinal and comparative approach in a representative sample of the Swiss work force). Second, we validated the short form by relating it to the performance in a ridicule and teasing scenario test. As it was shown previously that gelotophobes do not distinguish well between teasing and ridicule. We aimed to replicate this well-established finding in order to show the validity of the PhoPhiKat-9. Moreover, we established first relations of the three dispositions to relevant work place related variables (global life satisfaction, work satisfaction, work stress) in a large-scale representative sample of Swiss employees, to see whether the dispositions could help explaining vulnerabilities in the work place.

## Method

### Participants

#### Construction sample

The sample consisted of 157 German-speaking adults (34 males, 123 females). The age ranged between 18 and 59 years old (*M* = 28.l2, *SD* = 9.34).

#### Replication sample 1

The sample consisted of 1774 German-speaking adults (443 males, 1331 females). The age ranged between 18 and 79 years old (*M* = 38.44, *SD* = 12.41).

#### Validation sample 1

The sample consisted of 246 German-speaking adults (204 females, 42 males). The age ranged between 19 and 72 years old (*M* = 42.54, *SD* = 12.66).

#### Validation sample 2

The sample consisted of 1248 German-speaking adults (627 males, 627 females) from the NCCR- LIVES (data from the first wave of data collection in 2012). The age ranged between 26 and 56 years old (*M* = 42.73, *SD* = 8.73). The sample is representative for the Swiss working population.

### Instruments

The *PhoPhiKat-45* (Ruch and Proyer, 2009) is a 45-item questionnaire for the assessment of gelotophobia (a sample item is “When they laugh in my presence I get suspicious”), gelotophilia (“When I am with other people, I enjoy making jokes at my own expense to make the others laugh”), and katagelasticism (“I enjoy exposing others and I am happy when they get laughed at”). Answers are given on a four-point answer format (1 = strongly disagree to 4 = strongly agree). Ruch and Proyer (2009) reported high reliability coefficients (all alphas ≥0.84) and high retest-reliabilities ≥0.77 and ≥0.73 for a 3 and 6-month time period, respectively.

The *Ridicule Teasing Scenario Questionnaire Revised* (RTSqr; Platt, 2008) contains nine scenarios that assess emotions toward predetermined ridicule and teasing social scenarios. Four teasing, four ridicule, and one ambiguous scenarios are presented with short stories where participants rate to which extent they would experience eight emotions (joy, sadness, anger, disgust, surprise, shame, and fear plus contempt in the revised version) on a nine point Likert scale (from 0 = lowest to 8 = highest experience of emotions). Eight total scores are computed for both ridicule and teasing by averaging across the four scenarios.

The *Satisfaction with Life Scale* (SWLS; Diener et al., 1985) assesses the participants' life satisfaction. Answers are given on a seven-point scale (1 = strongly disagree to 7 = strongly agree). A sample item is “The conditions of my life are excellent.” In the current study (Validation Sample 2), the Cronbach's alpha was high (α = 0.89).

*Global work satisfaction* was assessed by one item (“In general, how satisfied are you with your work?”) on a four-point scale (1 = not satisfied at all to 4 = very satisfied).

The *General Work Stress Scale* (GWSS; De Bruin, 2006) is a nine item questionnaire assessing individually perceived demands of the workplace (e.g., “Do you become so stressed at work that you forget to do important tasks”). A five-point answer format is used (1 = never to 5 = always) measuring work stress as a one-dimensional construct. Cronbach's alpha in the current study (Validation Sample 2) was .87 and thus comparable to earlier findings (see De Bruin, 2006).

### Procedure

#### Participant recruitment

Participants were recruited in four independent surveys, three online surveys, and one mixed-method survey. They were not paid, but were offered an individual feedback on their personality scores (on demand) or could receive a gift voucher/make a donation in Validation Sample 2. All participants stayed anonymous at all times and they were free to withdraw from the study at any time. The studies fulfilled the ethical standards for research of the APA and approval from local ethic committees was granted.

#### Construction sample

The study was announced on the website of the University of Zurich and in a free local newspaper distributed in the public transport of the Zurich area. Participants received a link to the online survey and filled in the questionnaires.

#### Replication sample

Participants completed the survey on a website for research purposes hosted by the lab of the authors (http://www.charakterstaerken.org). The website was promoted by different means, such as press coverage (e.g., newspapers articles) and by contacting specific occupational groups, in order to ascertain heterogeneity of the sample.

#### Validation sample 1

Individuals from the Replication Sample were contacted via email approximately 10-month after their initial participation and invited to take part in a new online survey. In this online survey, the participants completed the PhoPhiKat-9 short-form (plus one item) and the RTSqr.

#### Validation sample 2

The data was collected within NCCR- LIVES (Swiss National Centre of Competence in Research LIVES—Overcoming vulnerability: Life course perspectives; data from the first wave of data collection in 2012). A representative sample of participants was drawn from the Swiss National Register of Inhabitants. In a mixed-method design, participants completed a first part of a questionnaire by phone or online (socio-demographic data and employment information), and the second part of the questionnaire online or paper-pencil (including the PhoPhiKat-9, SWLS, GWSS).

#### Ethics statement

This study complies with the ethical standards of the Swiss Society for Psychology. Also, the study was approved by the Ethics Committee of the Institute of Psychology, University of Zurich. All participants gave consent to participate and were free to withdraw from the study at any time, and their anonymity was ensured. As incentive, they could receive a personalized feedback in the *Construction Sample*, the *Replication Sample*, and the *Validation Sample 1*. Additionally, for *Validation Sample 2*, the institute that conducted the data collection obtained informed consent, kept the personal information, and researchers received a dataset without any personal information, in which participants were assigned numerical codes. Participants were compensated for their participation with a gift for a value of 20 Swiss francs.

#### Construction of the short form PhoPhiKat-9

The items for the PhoPhiKat-9 were selected from the PhoPhiKat-45 (Ruch and Proyer, 2009) in the Construction Sample. For the gelotophobia scale, items were selected to represent three facets found by Platt et al. (2012); i.e., (a) coping with derision (i.e., “I avoid showing myself in public because I fear that people could become aware of my insecurity and could make fun of me”); (b) disproportionate negative responses to being laughed at (“It takes me very long to recover from having been laughed at”); and (c) paranoid sensitivity to anticipated ridicule (“When strangers laugh in my presence I often relate it to me personally”). The selected items had the highest factor loading on each facet respectively (Construction Sample; cf. Platt et al., 2012).

For selecting the items for gelotophilia and katagelasticism, a principal component analysis was computed with the 45 items of the PhoPhiKat-45. Three component were extracted and rotated according to the Oblimin criterion (delta = 0). The components represented the three traits and were labeled accordingly. The rationale for the selection of the items was: (a) highest factor loading on the intended factor (and low secondary loadings; the difference between secondary loadings should be ≥0.30), (b) high corrected item-total correlations, and (c) the content should not overlap too strongly with the items that were already selected. Table 1 shows descriptive statistics for the nine item short form^3^.

**Table 1:** **Descriptive statistics and factor loadings for the nine items of the PhoPhiKat-9 short form in the replication sample**.

**Item description**	**Scale**	***M***	***SD***	**CITC**	**Loadings**		
					**Gelotophobia**	**Gelotophilia**	**Katagelasticism**
“Public attention” (4)	Pho	2.22 (2.23)	0.92 (0.96)	0.53 (0.66)	**0.71 (0.63)**	−0.16 (−0.22)	0.12 (0.01)
“No difference” (8)	Phi	2.28 (2.11)	0.90 (0.95)	0.28 (0.48)	**0.59 (**−**0.20)**	0.18 (0.49)	0.11 (−0.05)
“Laughing at others (3)	Kat	1.36 (1.35)	0.63 (0.63)	0.52 (0.37)	−0.05 (−0.06)	−0.02 (−0.07)	**0.81 (0.49)**
“Self-focus” (7)	Pho	1.93 (2.11)	0.84 (0.90)	0.49 (0.62)	**0.79 (0.62)**	0.25 (−0.14)	0.12 (−0.14)
“Fun maker” (14)	Phi	2.10 (2.23)	0.91 (0.95)	0.40 (0.56)	−0.05 (0.07)	**0.88 (0.63)**	−0.02 (0.16)
“Causing fights” (6)	Kat	1.60 (1.43)	0.74 (0.66)	0.42 (0.44)	−0.11 (0.12)	0.06 (0.09)	**0.68 (0.50)**
“Long recovery” (25)	Pho	2.21 (2.21)	0.90 (1.11)	0.52 (0.40)	**0.79 (0.37)**	−0.03 (−0.23)	−0.12 (0.17)
“No shame” (26)	Phi	2.21 (2.07)	0.89 (0.93)	0.44 (0.70)	0.12 (0.02)	**0.76 (0.74)**	0.04 (0.07)
“Part of life” (27)	Kat	1.69 (1.77)	0.79 (0.89)	0.45 (0.56)	0.15 (−0.05)	−0.04 (−0.05)	**0.80 (0.68)**
Cronbach's α					0.70 (0.88)	0.56 (0.87)	0.66 (0.84)
*M*					2.12 (1.97)	2.20 (2.43)	1.55 (1.99)
*SD*					0.70 (0.54)	0.66 (0.55)	0.55 (0.46)

*N = 1774. M, Mean; SD, Standard Deviation. Item descriptions refer to paraphrases. CITC, corrected item total correlation; Pho, gelotophobia. Phi, gelotophilia; Kat, katagelasticism. First column numbers in brackets are corresponding to position of the item on the PhoPhiKat-45. Bold values indicate high loadings*.

Next, we examined the factor structure of the nine item short form in a principal component analysis in the Replication Sample. Three components were extracted (Eigenvalues were 2.50, 2.09 and 0.92, respectively; explained variance = 61.19%) and rotated to the Oblimin criterion (delta = 0). Component 1 contained all gelotophobia items plus one item (with a high negative loading) that originally belonged to the gelotophilia scale (with loadings ranging from −0.59 to .79; see Table 1), component 2 constituted of the katagelasticism items (loadings ranging from 0.68 to 0.81), and component 3 of the remaining two gelotophilia-items (loadings were 0.76 and 0.88; see Table 1). Thus, eight items had their highest loadings on their target component, as theoretically expected, and had no high loadings on the other two components. However, one gelotophilia-item (“There is no difference for me whether people laugh at me or laugh with me”) had its highest loading on the gelotophobia factor.

Investigating the nature of the short form, we computed a confirmatory factor analysis (CFA) for three different models (Replication Sample, *N* = 1,774). To evaluate the model fit, RMSEA and SRMR values lower than 0.10 were assumed to indicate acceptable fit (e.g., Browne and Cudeck, 1993). According to Bollen and Long (1993), a RMSEA of 0.09 SRMR of 0.06 would be around the limit of being a reasonable error. We further followed the recommendations of Schermelleh-Engel et al. (2003), additionally reporting CFI and TLI. For model 1, we assumed correlated factors and loadings of each item on one factor alone, without secondary loadings on another factor. The null hypothesis of perfect fit for this model was rejected [$\chi_{\left(24\right)}^{2}$ = 496.54, *p* < 0.001; CFI = 0.85, TLI = 0.77, RMSEA = 0.105 (0.097–0.114), SRMR = 0.08]. For model 2, the assumptions were the same as for model 1 except for the first gelotophilia-item, which was allowed to have a second loading on the gelotophobia-factor. This model yielded better results [$\chi_{\left(23\right)}^{2}$ = 301.99, *p* < 0.001; CFI = 0.91, TLI = 0.87, RMSEA = 0.083 (0.075–0.091), SRMR = 0.06] with acceptable (but not high) model fit indices. In model 3, the gelotophilia-item was allowed to load only on the gelotophobia factor, with the loading on the gelotophilia factor restricted to zero, while the other model specifications remained the same. The model fit was acceptable [$\chi_{\left(24\right)}^{2}$ = 357.24, *p* < 0.001; CFI = 0.89, TLI = 0.86, RMSEA = 0.088 (0.080–0.097), SRMR = 0.07]. Thus, model 2 and 3 yielded acceptable solutions, with one gelotophilia item also loading on the gelotophobia factor. As this item worked well in the earlier studies (see Ruch and Proyer, 2009) we therefore did not consider this a serious deviation.

## Validation results

### Characteristics of the PhoPhiKat–9 in the validation sample 1

First, the descriptive statistics of the PhoPhiKat-9 items in the Validation Sample 1 are reported in Table 2. Means, standard deviations, Cronbach's alpha and the corrected item-total correlations (CITCs) can be seen in Table 2.

**Table 2:** **Descriptive statistics of the PhoPhiKat-9 and PhoPhiKat-45 in the validation sample 1**.

	***M***	***SD***	**Alpha**	**CITC range**	***t*_(224)_**
**PhoPhiKat-9**					
Gelotophobia	2.25	0.73	0.70	0.41–0.52	8.46^***^
Gelotophilia	2.23	0.71	0.69	0.50–0.53	−8.24^***^
Katagelasticism	1.52	0.46	0.38	0.15–0.28	−16.14^***^
**PhoPhiKat-45**					
Gelotophobia	1.99	0.56	0.89	0.25–0.69	
Gelotophilia	2.47	0.55	0.89	0.45–0.68	
Katagelasticism	1.97	0.43	0.84	0.27–0.63	

*N = 201–246. M, Mean; SD, Standard Deviation; Alpha, Cronbach's alpha; CITC, corrected item-total correlation; t-tests (df = 224) for mean level differences of the short and long form scales of gelotophobia, gelotophilia, and katagelasticism*.

*** *p < 0.001*.

The corrected item-total correlations (CITC) ranged between *r* = 0.15 and *r* = 0.53 for the short form. For the katagelasticism scale, the CITCs were remarkably lower and all below *r* = 0.30 (see Table 2). The Cronbach's alpha coefficients of gelotophilia and gelotophobia were acceptable (0.69 and 0.70; see Table 2), while the alpha of the katagelasticism scale was low (0.38). As expected, the Cronbach's alpha coefficients were smaller in the short form than in the PhoPhiKat-45 (see Table 2) due to the smaller number of items.

Second, we investigated mean level differences between the gelotophobia, gelotophilia, and katagelasticism scale, assessed by the short and the long form with *t*-tests for dependent samples. As shown in Table 2, the gelotophobia and gelotophilia means were higher in the short form, compared to the long form. The mean score of katagelasticism was lower in the short form assessment than the long form. Importantly, the results indicated that the cut-off for gelotophobia (>2.5 in the gelotophobia scale of the PhoPhiKat-45) could not be applied in the short form, as this would lead to an over-estimation of gelotophobes due to the increased mean in the short form. Therefore, we estimated the cut-off score equivalents for the short form by means of plotting the gelotophobia scores of the short and long form in a bivariate plot. The plot indicated that the equivalent of the 2.5 cut-off in the long form was reached by the approximate cut-off score of 2.67 in the short form. In both samples, the gelotophobia scores reached a cumulative percentage of 80.9% at the values of 2.47 (long form) and 2.67 (short form). With this cut-off equivalent, that classification of gelotophobes was only minimally different between the PhoPhiKat-45 and the PhoPhiKat-9. Splitting the group according to the criterion of the long form (cut-off of 2.5) resulted in 40 individuals being classified as gelotophobes. Splitting the group according to the cut-off equivalent in the ultra-short form (>2.67) resulted in 43 individuals being classified as gelotophobes^4^. Third, we investigated the correlations of the short and the long form of the PhoPhiKat. The correlations between the respective traits of the short and long form were high (0.58–0.76, *p* < 0.001). As expected (see Ruch and Proyer, 2009), both gelotophobia scales were unrelated to the katagelasticism scales (−0.03 to −0.10, n.s.) and negatively related to the gelotophilia scales (−0.36 to −0.42, *p* < 0.001). The katagelasticism scales were positively related to gelotophilia (0.34–0.38, *p* < 0.001). Previously reported correlation patterns could be replicated for both forms of the PhoPhiKat and the inter-correlations between the short and long form indicated an acceptable content overlap^4^.

### Predicting responses toward ridicule and teasing scenarios

**Figure 1:**
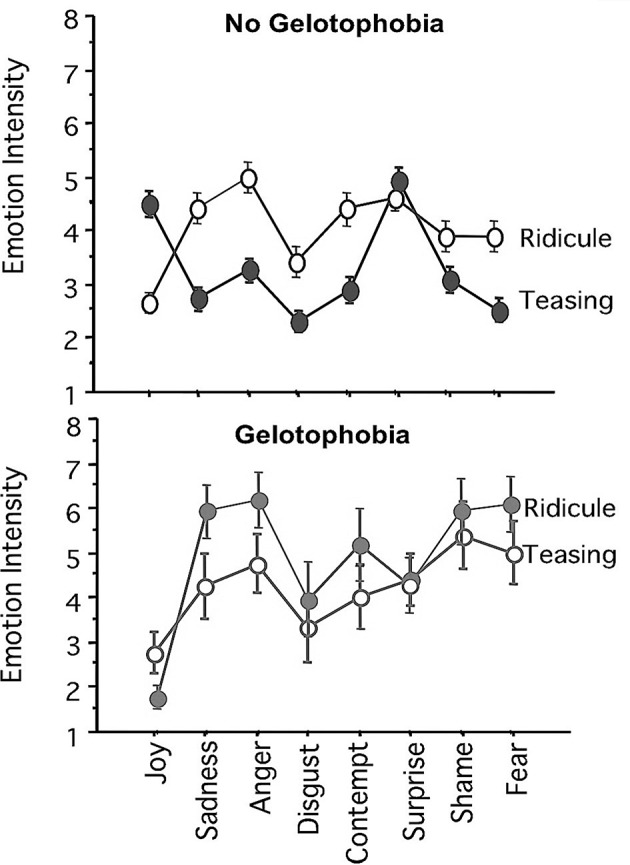
**Means and confidence intervals (95%) of the eight emotion ratings toward ridicule scenarios and teasing scenarios in gelotophobes and individuals with no fear of being laughed at (no gelotophobia)**.

To investigate the criterion validity of the PhoPhiKat-9, we utilized the RTSqr in the Validation Sample 1. Earlier research (e.g., Platt, 2008) showed that gelotophobes did not distinguish between ridicule and teasing when having to rate the emotions toward ridicule and teasing scenarios, assigning predominantly low joy, high fear, and high shame to both kinds of scenarios. Thus, in a first step, we investigated whether individuals above the cut-off point for gelotophobia would show similar response patterns of feeling high negative emotions and low joy when confronted with ridicule and teasing scenarios. We applied the cut-off equivalent for the short form (no gelotophobia ≤2.67, *n* = 203; gelotophobia >2.67, *n* = 43 individuals) for gelotophobia and computed two repeated measures ANOVAs (for the ridicule and teasing scenarios), with gelotophobia group (no gelotophobia vs. gelotophobia) as factor, the eight emotion ratings as repeated measures, and the intensity of emotion as dependent variable. For the ridicule scenarios, results showed that both main effects for type of emotion [*F*_(7, 1393)_ = 65.61, *p* < 0.001, $\eta_{\text{p}}^{2}$ = 0.248] and gelotophobia group [*F*_(1, 199)_ = 16.73, *p* < 0.001, $\eta_{\text{p}}^{2}$ = 0.078] were significant. Furthermore, the results were qualified by an interaction between gelotophobia group and type of emotion, *F*_(7, 1393)_ = 16.86, *p* < 0.001, $\eta_{\text{p}}^{2}$ = 0.078. Figure 1 shows the means and confidence intervals (95%) of the eight emotion ratings in the two groups (gelotophobia vs. no gelotophobia) toward ridicule and teasing scenarios.

Replicating the findings of Platt (2008), both groups of individuals assigned ridicule to negative feelings (mainly anger) and low joy. Figure 1 shows that the gelotophobes had higher ratings of sadness, anger, disgust, contempt, shame, and fear compared to individuals without a fear of being laughed at (all *p* < 0.05, Bonferroni corrected). In line with the predictions, the level of gelotophobia predicted the disproportionate negative responses to being laughed at by eliciting more intense negative feelings toward ridicule scenarios.

Concerning the teasing scenarios, results showed that both main effects for type of emotion [*F*_(7, 1400)_ = 27.67, *p* < 0.001, $\eta_{\text{p}}^{2}$ = 0.094] and gelotophobia group [*F*_(1, 200)_ = 27.40, *p* < 0.001, $\eta_{\text{p}}^{2}$ = 0.121] were significant. Furthermore, the results were qualified by an interaction between gelotophobia group and type of emotion, *F*_(7, 1400)_ = 33.61, *p* < 0.001, $\eta_{p}^{2}$ = 0.144. As Figure 1 indicates, gelotophobes were higher in anger, fear, disgust, contempt, shame, and lower in joy and surprise, compared to individuals with no fear of being laughed at (all *p* < 0.05, Bonferroni corrected). Thus, gelotophobes did not evaluate the friendly teasing scenarios as such, but assigned them negative emotions (mostly shame, fear, and anger) and low joy, just as to the ridicule scenarios. This replicates former findings (see Platt, 2008) but also shows that this effect can be found for the short form of the PhoPhiKat-9 as well, validating its suitability for the assessment of gelotophobia. Furthermore, the results show the bias of gelotophobes toward social situations in which teasing occurs (i.e., banter at work, pro-social teasing among friends): Instead of seeing the joyful component, gelotophobes report that they would mainly feel anger, shame, and fear.

Next, we investigated the role of katagelasticism and gelotophilia in predicting responses to ridicule and teasing. As no cut-offs exists for these dispositions, we decided to compute four hierarchical multiple regression analyses (two for the teasing and ridicule scenarios) with gelotophilia and katagelasticism as criteria and the eight emotion ratings as predictor variables. A multiple regression model was estimated in which predictors were entered when they added to the prediction of the dependent variable substantially or removed, when they did no longer add substantially to the prediction due to the inclusion of another variable (STEPWISE-procedure). These predictors entered the analysis in a second block preceded by age and gender in a first block which entered simultaneously. First, the findings on gelotophilia are reported. In the ridicule scenarios, the regression led to a multiple correlation coefficient of *R* = 0.36, *F*_(3, 197)_ = 9.62, *p* < 0.001. Gelotophilia was solely predicted by the assigned joy to the scenarios (β = 0.19, *p* < 0.001), while neither age (β = −0.003, *p* = 0.445) nor gender (β = −0.13, *p* = 0.282) had a significant contribution. No other emotion rating entered in a further step. In the teasing scenarios, the multiple correlation was *R* = 0.47 [*F*_(3, 198)_ = 18.95, *p* < 0.001]. Again, gelotophilia was predicted by the joy rating entering the equation (β = 0.36, *p* < 0.001), while neither age (β = −0.08, *p* = 0.239) nor gender (β = −0.06, *p* = 0.331) contributed significantly. No other variable entered the equation. As expected, joy mainly predicted gelotophilia in both types of scenarios.

Concerning the prediction of katagelasticism in teasing scenarios, gender turned out to be significant predictor in the first step, *F*_(2, 198)_ = 3.26, *p* = 0.037, *R* = 0.18, β = −0.20, *p* = 0.022. Age did not predict the katagelasticism score, β = −0.01, *p* = 0.162. No further variable entered the equation, indicating that none of the emotion ratings toward teasing scenarios were good predictors of the joy of laughing at others. For the ridicule scenarios, the regression led to a multiple correlation coefficient of *R* = 0.34 [*F*_(4, 196)_ = 6.25, *p* < 0.001]. Gender (entering in the first step) had a significant contribution (β = −0.18, *p* = 0.030), but not age (β = −0.01, *p* = 0.059). Furthermore, there were unique contributions of the self-reported joy in ridicule (β = 0.10, *p* < 0.001) and contempt to the prediction of katagelasticism (β = 0.04, *p* = 0.009).

### Gelotophobia, gelotophilia, and katagelasticism and workplace outcomes

Next, we investigated the relationship of the three dispositions toward ridicule and laughter to life and global work satisfaction, as well as work stress in a large and representative sample of Swiss employees (Validation Sample 2). This could give first indication of whether the three dispositions can help explaining workplace related vulnerabilities. Findings for gelotophobia are presented first. Here, the established cut-off score warrants the analysis of gelotophobes vs. non-gelotophobes. We utilized the adapted cut-offs for the PhoPhiKat-9. The means and standard deviations can be seen in Table 3; for individuals with (gelotophobia group; scores >2.67; *n* = 115) and without a fear of being laughed at (no gelotophobia; scores ≤2.67; *n* = 1017) separately^5^.

**Table 3:** **Group differences for individuals with or without gelotophobia in life satisfaction, global work satisfaction, and work stress**.

	**No gelotophobia**		**Gelotophobia**			
	***M***	***SD***	***M***	***SD***	***F*_(1, 1130)_**	**$\eta_{\text{p}}^{2}$**
Life satisfaction	5.40	1.04	4.56	1.26	68.04^***^	0.06
Work satisfaction	3.36	0.58	3.18	0.50	11.63^***^	0.01
Work stress	1.81	0.54	2.14	0.76	37.43^***^	0.03

*No Gelotophobia, Gelotophobia scores ≤ 2.67 on the PhoPhiKat-9. Gelotophobia, Gelotophobia scores >2.67 on the PhoPhiKat-9*.

*** *p < 0.001*.

Table 3 shows the means in life satisfaction, global work satisfaction, and work stress in individuals with or without a fear of being laughed at. Investigating group differences, we computed three ANOVAs with the gelotophobia group as the factor and life satisfaction, work satisfaction, and general work stress as dependent variables. Results indicated gelotophobes reported lower levels of life satisfaction and global work satisfaction, as well as higher perceived work stress (see Table 3) compared to individuals with no fear of being laughed at. Thus, in line with our hypotheses, gelotophobia was negatively related to indicators of satisfaction and went along with higher reported stress.

For the investigation of the relationship of gelotophilia and katagelasticism to life and work satisfaction and work stress, we computed hierarchical multiple regression analysis with gelotophilia and katagelasticism as predictors and life satisfaction, work satisfaction, and work stress respectively as criteria. The predictors entered the analysis simultaneously in a second block preceded by age and gender in a first block (both entering simultaneously as well). To predict life satisfaction, the regression led to a multiple correlation coefficient of *R* = 0.12, *F*_(4, 1246)_ = 4.16, *p* = 0.002. Life satisfaction was predicted by gelotophilia (β = 0.07, *p* = 0.022), and katagelasticism (β = −0.10, *p* < 0.001), while neither age (β = 0.05, *p* = 0.059) nor gender (β = 0.007, *p* = 0.800) had a significant contribution. For work satisfaction, the multiple correlation was *R* = 0.06 [*F*_(4, 1238)_ = 0.96, *p* = 0.431]. None of the predictors had a significant contribution (all *p* > 0.200). For work stress, the multiple correlation was *R* = 0.14 [*F*_(4, 1236)_ = 6.03, *p* < 0.001). Only katagelasticism predicted work stress (β = 0.14, *p* < 0.001), while neither gelotophilia (β = −0.01, *p* = 0.708), age (β = 0.03, *p* = 0.370) nor gender (β = −0.01, *p* = 0.672) contributed significantly.

## Discussion

The aim of this study was two-fold. First, we adapted the PhoPhiKat-9 for the use in large-scale studies and as a screening tool in applied settings. Second, we established first relations to work-related outcome variables in a representative sample of the Swiss work force. In terms of construction of the PhoPhiKat-9, all three dispositions can be reliably assessed with this ultra-short form. The psychometric characteristics were satisfactory when considering that this ultra-short form should only be used in large samples. The relations to demographic variables were comparable to relations found for the standard PhoPhiKat-45. Two deviations from the original PhoPhiKat-45 occurred: First, the cut-off point for gelotophobia set at 2.5 on the original PhoPhiKat-45 could not be utilized with the ultra-short form, as the means were generally higher compared to those of the original scale. We therefore estimated cut-off score equivalents basing on the criterion for the sample that had filled in both forms (long and short form). The new cut-off was set at 2.67. Second, one item representing gelotophilia revealed high loadings on the gelotophobia component as well, which may need consideration in future studies (i.e., re-phrasing item).

We utilized two independent samples to validate the PhoPhiKat-9. In line with former studies (Platt, 2008; Platt et al., 2009), the present results replicated the misperception of teasing and ridicule by individuals with elevated scores in gelotophobia. In a work based context, gelotophobes are probably going to have problems distinguishing between the friendly smiling and banter between colleagues (see also Hofmann et al., 2015), taking it for bullying. There is a stable pattern of reporting being a victim of bullying and greater expressions in gelotophobia already starting from the age of six (self- and peer-reports; for an overview see Ruch et al., 2014). Gelotophobes are therefore more likely to feel bullied and discriminated in the workplace, leading to more perceived stress, and lower satisfaction with work and life (cf. Proyer et al., 2012b). This was substantiated by findings of the second validation, where gelotophobes described themselves as less satisfied with life and work, as well as more stressed at the work place, compared to those individuals without gelotophobia.

With respect to gelotophilia, the main finding was that higher ratings of gelotophilia went along with higher ratings of joy toward both, teasing and ridicule scenarios in the RTS-qr. Gelotophiles take humorous instances light-heartedly and will initiate them with pleasure. Surprisingly, no relations of gelotophilia to satisfaction and work stress were found, indicating that other factors might be more important in the prediction of those outcomes. Interestingly, katagelasticism was predicted by the joy and contempt assigned to ridicule scenarios. In line with the descriptions by Ruch and Proyer (2009), katagelasticists get pleasure from laughing at other and will also use this as a social corrective, or to take revenge on others (i.e., “an eye for an eye,” see Ruch and Proyer, 2009). Already Tomkins (1969) stated that contempt toward another person might lead to laughter directed at this individual (see Hofmann et al., 2015): Katagelasticists might ridicule a person that is disliked or has overstepped a norm, and the ridicule goes along with laughter and humor targeted at the person (e.g., Tomkins, 1969; “the laugh becomes a vehicle of contempt,” p. 367). Unexpectedly, the Cronbach's Alpha of the katagelasticism scale was lower in this sample than in the other three samples (0.38 compared to 0.64, 0.65, and 0.65 in the construction and validation samples respectively). Thus, the findings on the katagelasticism scale are best treated more cautiously in this sample, while the scale is stable in the other three samples. With respect to the second validation, negative relations of katagelasticism to life satisfaction and positive relations to work stress were found. One possible explanation might be that katagelasticists generally experience more conflicts with others (generally, as well as in the work place), as they overtly laugh at them. This potentially could lead to problems in the work place and consequently to increasing levels of stress. Alternatively, katagelasticism has been shown to positively relate to psychoticism and psychopathic traits (see Proyer et al., 2012a), as well as lower social desirability. Those higher order traits might be (partially) responsible for more conflicts that could lead lowered life satisfaction and higher work stress. Thus, future studies may investigate this hypothesized mechanism and also investigate the incremental validity of katagelasticism compared to higher order traits, such as psychoticism.

Two main limitations prevail: The factor structure of the PhoPhiKat-9 did not reveal a consistent pattern for the gelotophilia scale. The item “There is no difference for me whether people laugh at me or laugh with me” loaded higher on the gelotophobia scale than on the gelotophilia scale. It is hypothesized that this item was maybe interpreted differently to the initial meaning: If individuals fear being laughed at, it does not make a difference to them if people laugh with or at them, as both is negative. In the original sense, the item possessed a positive connotation: It does not make a difference whether people laugh at or with a gelotophile, as both is equally enjoyable. This item needs a clearer phrasing toward all laughter being good to fit on the gelotophilia factor. Moreover, the mechanisms between gelotophobia, and the lowered satisfaction and work stress need to be looked at in more detail, at best by studying phenomena longitudinally. Furthermore, future studies should aim at investigating the incremental validity of the three dispositions toward ridicule and laughter in the prediction of workplace related outcomes when controlling for broader personality traits (i.e., the “Big Five”). Moreover, future studies may opt for more balanced samples in terms of gender ratio.

## Application

In light of work place behavior and career trajectories, all three dispositions relate to relevant behaviors and perceptions, such as work place bullying and perceived discrimination (e.g., Platt, 2008; Platt et al., 2009; Ruch and Proyer, 2009; Proyer and Ruch, 2010; Chen and Liu, 2012). The measurement of gelotophobia, gelotophilia, and katagelasticism in work place environments can indicate important team processes relating to the popular topics of “good work practice” and “avoidance of incidents of work place bullying.” Gelotophobia may link to unfavorable work outcomes, like feeling one is being bullied, misunderstanding any laughter and humor in teams, and maybe being more stressed and less satisfied with the work environment as a consequence. Understanding the (mis-) perception will assist in redressing the bias often placed toward the alleged victims. This is of concern not only to institutions, human resource units and those practicing workplace law but also to public and governmental bullying initiatives. Hence, intervention programs should aim at raising awareness about the role of laughter and laughing at the workplace in general, but also those with greater fear of being laughed at directly. There are no standardized programs addressing the fear of being laughed at, but learning about humor and laughter and how to deal with (perceived) ridicule may be beneficial for those with extreme expressions, i.e., formulating guidelines and offering advice for applied psychologists (see Platt et al., 2012). The ultra-short form is only utilized for screening larger samples, yet, the judgments on the three dispositions need to be consolidated by giving the PhoPhiKat-45 (or the short form PhoPhiKat-30) to individuals that potentially fear being laughed at or potentially are work place bullies. This potentially helps to improve team processes and relations among co-workers and customers.

## Ethics statement

This study was carried out in accordance with the recommendations of “Swiss Psychological Association”; and the Ethics Committee of the Department of Psychology, University of Zurich, with written informed consent from all subjects. All subjects gave written informed consent in accordance with the Declaration of Helsinki.

## Author contributions

Conception or design of the work: WR and JH. Data collection: JH, WR, RP, TP, FG. Data analysis and interpretation: JH, WR, RP, and FG. Drafting the article: JH. Critical revision of the article: WR, RP, FG, and TP. Final approval of the published version JH, WR, RP, FG, TP.

## Funding

This publication benefited from the support of the Swiss National Centre of Competence in Research LIVES—Overcoming vulnerability: Life course perspectives, which is financed by the Swiss National Science Foundation (grant number: 51NF40-160590). The authors are grateful to the Swiss National Science Foundation for its financial assistance.

### Conflict of interest statement

The authors declare that the research was conducted in the absence of any commercial or financial relationships that could be construed as a potential conflict of interest.

## Footnotes

^1^Weiss et al. (2012) could show that gelotophobes showed deficits in handling their emotions, more self-reported aggressive behavior, and anger proneness.

^2^We followed the guidelines recommended by Smith et al. (2000). One requirement is that the original instrument has shown enough evidence of reliability and validity. For the PhoPhiKat-45, a variety of validation studies have shown its good psychometric characteristics and validity (see Ruch et al., 2014 for a review). A further requirement suggests that the development of the short form and the analysis of its psychometric properties should be conducted in two independent samples. We included data of two independent samples for the construction and replication, as well as two samples for the validation.

^3^As different samples were utilized, internal consistencies are reported for all samples separately: In the replication sample, the Cronbach's alpha of the PhoPhiKat-9 were 0.69 for gelotophobia, 0.57 for gelotophilia, and 0.64 for katagelasticism. In the validation sample 1, the Cronbach's alpha of the PhoPhiKat-9 were 0.70 for gelotophobia, 0.69 for gelotophilia, and 0.38 for katagelasticism. In the validation sample 2, the Cronbach's alpha of the PhoPhiKat-9 were 0.64 for gelotophobia, 0.54 for gelotophilia, and 0.65 for katagelasticism.

^4^For the PhoPhiKat-45, age and gender differences were reported (see Ruch and Proyer, 2009). For replication purposes, we computed correlations between the three dispositions, age and gender in both forms. Concerning the relations to the participant's age, both katagelasticism scales were negatively related to age, as previously found; *r*_short_ (225) = −0.11, *p* = 0.106, *r*_long_ (246) = −0.14, *p* = 0.031. Also, the participant's age correlated negatively to gelotophobia [*r*_short_ (225) = −0.11, *p* = 0.103, *r*_long_ (246) = 0.18, *p* = 0.005], but was unrelated to gelotophilia [*r*_short_ (225) = −0.04, *p* = 0.564, *r*_long_ (246) = −0.01, *p* = 0.878]. Fourth, we computed a MANOVA with gender as factor and the scales of the PhoPhiKat long and short forms as dependent variables. The overall effect was significant, *F*_(6, 218)_ = 2.21, *p* = 0.043, = 0.057. In line with the expectations, *post-hoc* tests indicated that males scored higher on both katagelasticism scales (*p* < 0.05). Males and females did not differ in gelotophobia and gelotophilia (all n.s.). The results show that the short form and long form revealed the same patterns of relationships to the demographic variables, replicating former findings (see Ruch and Proyer, 2009).

^5^Cut-off score equivalents for marked and extreme gelotophobia assessed with the short form are at 3.33 (marked) and 3.67 (extreme).
